# Supplementary material for: Plasma Sphingolipid Profile in Association with Incident Metabolic Syndrome in a Chinese Population-Based Cohort Study
Source: Nutrients. 2021 Jun 30;13(7):2263. doi: 10.3390/nu13072263 (PMC8308381; doi:10.3390/nu13072263)
Supplement: Supplementary file 1 [file nutrients-13-02263-s001.zip › nutrients-1267040-supplementary.pdf]

Table S1. Baseline plasma sphingolipids between incident MetS cases and non-cases.

Sphingolipids (mg/L)	CV (%)	Incident MetS		p Value
		No (n = 811)	Yes (n = 431)	
Ceramides				
Cer(d18:1/14:0) (10^{-2})	15.3	14.2 (12.3, 16.5)	13.8 (11.5, 16.5)	5.58×10^{-4}*
Cer(d18:1/16:0) (10^{-2})	17.3	47.3 (21.3, 105.2)	46.3 (17.3, 124.0)	6.89×10^{-2}
Cer(d18:1/18:0) (10^{-2})	19.1	30.1 (13.8, 65.9)	31.2 (11.4, 85.2)	3.02×10^{-1}
Cer(d18:1/18:1) (10^{-2})	23.9	4.63 (4.00, 5.37)	4.96 (4.08, 6.04)	1.43×10^{-2}*
Cer(d18:1/20:0) (10^{-2})	18.9	11.2 (8.20, 15.1)	12.0 (8.50, 16.9)	9.02×10^{-4}*
Cer(d18:1/20:1) (10^{-2})	26.4	6.50 (5.47, 7.73)	6.92 (5.55, 8.63)	9.78×10^{-3}*
Cer(d18:1/22:0)	16.9	0.85 (0.83, 0.87)	0.93 (0.90, 0.96)	4.63×10^{-4}*
Cer(d18:1/22:1)	23.8	0.48 (0.48, 0.49)	0.52 (0.51, 0.53)	1.18×10^{-2}*
Cer(d18:1/24:0)	16.4	2.55 (2.36, 2.76)	2.73 (2.45, 3.03)	8.68×10^{-2}
Cer(d18:1/24:1)	17.4	2.87 (2.71, 3.04)	2.93 (2.71, 3.17)	1.26×10^{-1}
Cer(d18:1/26:0)	24.6	0.56 (0.55, 0.57)	0.56 (0.55, 0.58)	2.88×10^{-1}
Cer(d18:1/26:1) (10^{-2})	29.9	4.02 (3.50, 4.62)	4.00 (3.37, 4.75)	7.48×10^{-1}
dhCers				
Cer(d18:0/16:0) (10^{-2})	22.1	2.19 (2.08, 2.30)	2.13 (2.01, 2.27)	8.70×10^{-2}
Cer(d18:0/18:0) (10^{-2})	25.0	1.47 (1.40, 1.55)	1.52 (1.43, 1.61)	7.11×10^{-1}
Cer(d18:0/18:1) (10^{-2})	26.6	1.04 (0.99, 1.09)	1.09 (1.03, 1.17)	1.63×10^{-1}
Cer(d18:0/20:0) (10^{-2})	25.6	0.83 (0.80, 0.86)	0.88 (0.85, 0.92)	1.06×10^{-1}
Cer(d18:0/20:1) (10^{-2})	28.0	0.68 (0.67, 0.70)	0.72 (0.70, 0.74)	2.02×10^{-1}
Cer(d18:0/22:0) (10^{-2})	21.2	4.33 (3.73, 5.03)	4.71 (3.83, 5.80)	4.19×10^{-2}
Cer(d18:0/22:1) (10^{-2})	25.1	10.1 (7.01, 14.6)	11.1 (6.43, 19.1)	1.30×10^{-2}*
Cer(d18:0/24:0) (10^{-2})	20.2	10.8 (7.53, 15.4)	11.7 (7.12, 19.2)	3.00×10^{-2}
Cer(d18:0/24:1) (10^{-2})	21.6	9.16 (7.33, 11.4)	9.61 (6.84, 13.5)	1.54×10^{-2}*
SMs				
SM C34:0	19.2	16.0 (12.5, 20.4)	15.4 (11.1, 21.3)	9.13×10^{-6}*
SM C36:0	9.0	9.00 (6.90, 11.7)	9.53 (6.77, 13.4)	9.88×10^{-4}*
SM C38:0	15.7	53.5 (28.8, 99.3)	53.4 (22.2, 128.4)	2.92×10^{-1}
SM C40:0	7.6	49.5 (35.9, 68.4)	49.9 (30.9, 80.6)	1.23×10^{-2}*
SM C42:0	6.8	49.4 (38.0, 64.1)	49.5 (34.3, 71.5)	7.64×10^{-1}
SM C32:1	20.4	4.73 (4.23, 5.29)	4.90 (4.21, 5.70)	3.79×10^{-2}
SM C34:1	14.4	40.2 (25.4, 63.5)	39.4 (21.8, 71.2)	6.85×10^{-1}
SM C34:2	16.3	12.6 (10.5, 15.2)	12.8 (9.91, 16.4)	1.01×10^{-2}*
SM C36:1	14.0	14.2 (11.6, 17.3)	14.6 (11.2, 19.0)	2.73×10^{-1}
SM C36:2	17.1	7.67 (6.68, 8.80)	8.08 (6.67, 9.79)	1.11×10^{-1}
SM C38:1	19.3	45.5 (27.4, 75.7)	44.8 (16.7, 120.3)	6.58×10^{-2}
SM C40:1	14.1	44.2 (26.4, 74.1)	43.9 (20.0, 96.6)	3.86×10^{-1}
SM C42:1	6.0	45.7 (34.1, 61.1)	46.3 (28.5, 75.1)	9.67×10^{-5}*
SM C42:2	14.0	37.9 (24.9, 57.7)	37.2 (20.9, 66.5)	1.03×10^{-1}
SM C42:3	5.9	42.2 (31.9, 55.8)	42.7 (29.3, 62.0)	7.62×10^{-3}*
SM C44:1	19.0	3.23 (2.98, 3.51)	3.36 (3.07, 3.69)	1.65×10^{-1}

SM C44:3	10.7	6.37 (5.65, 7.18)	6.64 (5.60, 7.89)	6.24×10^{-3}*
SM (OH)s				
SM (OH) C32:2	21.2	3.38 (3.12, 3.66)	3.36 (3.02, 3.75)	9.75×10^{-4}*
SM (OH) C34:0	18.7	2.87 (2.72, 3.03)	2.76 (2.57, 2.98)	7.28×10^{-4}*
SM (OH) C34:1	23.9	1.38 (1.34, 1.43)	1.35 (1.29, 1.40)	2.46×10^{-5}*
SM (OH) C34:2	21.1	1.86 (1.79, 1.94)	1.83 (1.74, 1.93)	2.24×10^{-4}*
SM (OH) C36:1	8.2	7.26 (6.65, 7.92)	6.97 (6.26, 7.76)	8.55×10^{-6}*
SM (OH) C36:2	7.7	8.80 (7.78, 9.95)	8.71 (7.46, 10.2)	2.19×10^{-3}*
SM (OH) C36:3	10.2	9.29 (8.63, 10.0)	9.31 (8.37, 10.4)	9.06×10^{-1}
SM (OH) C38:1	19.1	4.42 (4.08, 4.79)	4.38 (4.00, 4.80)	1.75×10^{-1}
SM (OH) C38:2	5.9	11.7 (10.5, 13.1)	11.6 (10.0, 13.4)	3.32×10^{-5}*
SM (OH) C38:3	11.2	24.0 (20.1, 28.8)	24.1 (19.2, 30.2)	2.28×10^{-1}
SM (OH) C40:1	23.1	3.25 (2.98, 3.54)	3.40 (2.99, 3.86)	5.05×10^{-1}
SM (OH) C40:2	20.6	11.8 (9.12, 15.1)	12.2 (8.52, 17.5)	4.47×10^{-1}
SM (OH) C40:3	13.9	8.41 (7.48, 9.47)	9.03 (7.75, 10.5)	7.62×10^{-2}
SM (OH) C40:4	7.3	16.5 (14.2, 19.1)	16.4 (13.5, 20.0)	4.64×10^{-2}
SM (OH) C42:2	14.0	2.39 (2.31, 2.47)	2.38 (2.27, 2.48)	8.25×10^{-1}
SM (OH) C42:3	12.9	6.13 (5.60, 6.71)	6.14 (5.40, 6.98)	7.36×10^{-1}
SM (OH) C42:4	27.6	0.56 (0.55, 0.57)	0.56 (0.55, 0.57)	2.65×10^{-2}
SM (OH) C44:0	19.3	1.11 (1.06, 1.17)	1.05 (1.00, 1.10)	3.62×10^{-3}*
SM (OH) C44:1	22.2	1.21 (1.18, 1.23)	1.19 (1.16, 1.23)	3.47×10^{-2}
SM (OH) C44:3	22.1	1.85 (1.80, 1.90)	1.83 (1.77, 1.89)	5.44×10^{-2}
SM (2OH)s				
SM (2OH) C30:2	25.1	1.21 (1.17, 1.25)	1.24 (1.19, 1.30)	3.69×10^{-1}
SM (2OH) C32:1	23.6	2.26 (2.17, 2.37)	2.16 (2.04, 2.30)	1.32×10^{-5}*
SM (2OH) C34:1	9.7	14.2 (12.1, 16.7)	14.1 (11.5, 17.3)	7.86×10^{-1}
SM (2OH) C40:0	5.3	20.7 (19.4, 22.1)	20.6 (19.0, 22.5)	1.77×10^{-2}*
SM (2OH) C40:1	6.5	45.0 (36.4, 55.6)	45.1 (33.7, 60.4)	2.98×10^{-2}
SM (2OH) C42:4	16.1	4.91 (4.44, 5.44)	5.03 (4.46, 5.68)	3.52×10^{-2}
GSLs				
HexCer(d18:1/12:0) (10^{-2})	29.6	3.54 (3.17, 3.96)	3.46 (2.95, 4.05)	2.77×10^{-1}
HexCer(d18:1/16:0)	22.9	1.10 (1.07, 1.13)	1.06 (1.03, 1.10)	1.66×10^{-2}*
HexCer(d18:1/18:0)	26.0	0.60 (0.59, 0.61)	0.56 (0.55, 0.58)	1.13×10^{-3}*
HexCer(d18:1/20:0) (10^{-2})	25.5	0.16 (0.16, 0.16)	0.15 (0.15, 0.15)	5.68×10^{-3}*
HexCer(d18:1/20:1) (10^{-2})	26.9	0.10 (0.10, 0.10)	0.09 (0.09, 0.09)	1.21×10^{-3}*
HexCer(d18:1/22:0)	20.9	1.21 (1.17, 1.25)	1.15 (1.10, 1.20)	2.53×10^{-3}*
HexCer(d18:1/22:1)	25.2	0.66 (0.65, 0.67)	0.64 (0.62, 0.65)	3.51×10^{-2}
HexCer(d18:1/24:0)	21.6	1.58 (1.51, 1.66)	1.50 (1.41, 1.59)	1.18×10^{-3}*
HexCer(d18:1/24:1)	23.4	2.21 (2.09, 2.35)	2.04 (1.90, 2.19)	1.63×10^{-3}*
GlcCer(d18:0/24:0) (10^{-2})	27.6	6.24 (5.18, 7.53)	5.88 (4.59, 7.51)	3.10×10^{-3}*
GlcCer(d18:0/24:1) (10^{-2})	27.4	4.36 (3.84, 4.96)	4.12 (3.50, 4.85)	1.25×10^{-1}
LacCer(d18:1/20:1) (10^{-2})	27.9	2.62 (2.45, 2.80)	2.72 (2.50, 2.96)	3.67×10^{-1}

Values are shown as geometric means (95% CIs). *p* values were calculated adjusted for age, sex, region (Beijing or Shanghai), and residence (urban or rural). *, $p < 0.05$ after correction for multiple testing using the Benjamini–Hochberg method. Cer, ceramide; CV, coefficient of variation; dhCer, dihydroceramide; GlcCer, glucosylceramide; GSL, glycosphingolipid;

HexCer, hexosylceramide; LacCer, lactosylceramide; MetS, metabolic syndrome; SM, sphingomyelin; SM (OH), sphingomyelin with one additional hydroxyl; SM (2OH), sphingomyelin with two additional hydroxyls.

Table S2. Eigenvalues and variance explained by the seven extracted factors (PCA).

Factor	Eigenvalue	Variance explained (%)	Cumulative
Factor 1	26.90	35.40	35.40
Factor 2	7.32	9.63	45.02
Factor 3	6.67	8.78	53.80
Factor 4	4.91	6.47	60.27
Factor 5	3.41	4.49	64.76
Factor 6	2.67	3.51	68.27
Factor 7	2.18	2.86	71.13

PCA, principal component analysis.

Table S3. Description of the seven extracted factors (PCA).

Factor	Description	Components
Factor 1	Hydroxysphingomyelins, long-chain SMs, and GSLs	SM (OH) C32:2 SM (OH) C34:0 SM (OH) C34:1 SM (OH) C34:2 SM (OH) C36:2 SM (OH) C36:3 SM (OH) C38:2 SM (OH) C40:1 SM (OH) C40:2 SM (OH) C40:4 SM (OH) C42:4 SM (2OH) C30:2 SM (2OH) C32:1 SM C32:1 SM C34:0 SM C34:1 SM C34:2 SM C36:1 SM C36:2 Cer(d18:1/22:0) Cer(d18:0/24:0) Cer(d18:0/22:0) Cer(d18:1/24:0) HexCer(d18:1/24:0) Cer(d18:1/22:1) LacCer(d18:1/20:1) GlcCer(d18:0/24:0) HexCer(d18:1/18:0) HexCer(d18:1/22:0)
Factor 2	SMs	SM C34:0 SM C34:1 SM C38:0 SM C38:1 SM C40:0 SM C40:1 SM C42:0 SM C42:1 SM C42:3 SM (OH) C38:3 SM (2OH) C40:1
Factor 3	Ceramides and dhCers	Cer(d18:1/16:0) Cer(d18:1/18:0) Cer(d18:1/18:1) Cer(d18:1/20:0) Cer(d18:1/20:1) Cer(d18:1/24:1) Cer(d18:1/26:0) Cer(d18:0/16:0) Cer(d18:0/18:0) Cer(d18:0/20:0) Cer(d18:0/20:1) Cer(d18:0/24:1) SM C36:1 SM C36:2 SM C42:2 HexCer(d18:1/12:0) HexCer(d18:1/20:1)
Factor 4	GSLs	HexCer(d18:1/24:1) HexCer(d18:1/18:0) HexCer(d18:1/20:1) GlcCer(d18:0:24:0) HexCer(d18:1/22:1) HexCer(d18:1/22:0) GlcCer(d18:0:24:1) HexCer(d18:1/20:0) HexCer(d18:1/16:0) HexCer(d18:1/24:0) Cer(d18:1/16:0)
Factor 5	Very-long-chain SMs and hydroxysphingomyelins	SM (OH) C36:1 SM (OH) C44:0 SM (OH) C44:1 SM (OH) C44:3 SM (2OH) C34:1 SM (2OH) C42:4 SM C34:0 SM C36:0 SM C44:1 SM C44:3
Factor 6	Very-long-chain hydroxysphingomyelins	SM (OH) C42:3 SM (OH) C38:3 SM (OH) C36:3 SM (OH) C42:2 SM (OH) C44:3 SM (2OH) C40:0 SM (2OH) C42:4 SM C42:0 SM C44:1
Factor 7	Very-long-chain ceramides and dhCers	Cer(d18:1/22:0) Cer(d18:0/22:0) Cer(d18:1/24:0) Cer(d18:0/24:0) Cer(d18:1/22:1) Cer(d18:0/22:1) SM C42:2

Cer, ceramide; dhCer, dihydroceramide; GlcCer, glucosylceramide; GSL, glycosphingolipid; HexCer, hexosylceramide; LacCer, lactosylceramide; PCA, principal component analysis; SM, sphingomyelin; SM (OH), sphingomyelin with one additional hydroxyl; SM (2OH), sphingomyelin with two additional hydroxyls.

Table S4. Association between baseline sphingolipid factors (PCA extracted) and incident metabolic syndrome components.

	Central obesity (n = 1156)		Hypertriglyceridemia (n = 1713)		Low HDL-cholesterol (n = 1312)		Elevated blood pressure (n = 702)		Hyperglycemia (n = 1345)	
	**RR (95% CI)**	***p***	**RR (95% CI)**	***p***	**RR (95% CI)**	***p***	**RR (95% CI)**	***p***	**RR (95% CI)**	***p***
Factor 1 (Hydroxysphingomyelins, long-chain SMs, and GSLs)										
Model 1	0.84 (0.74, 0.96)	0.011	0.89 (0.81, 0.98)	0.019	0.80 (0.67, 0.95)	0.010	0.90 (0.80, 1.01)	0.067	0.96 (0.93, 1.00)	0.081
Model 2	0.87 (0.75, 1.00)	0.058	0.93 (0.84, 1.03)	0.16	0.81 (0.67, 0.97)	0.020	0.86 (0.76, 0.97)	0.017	0.98 (0.94, 1.02)	0.36
Model 3	0.89 (0.77, 1.03)	0.12	0.92 (0.83, 1.02)	0.13	0.80 (0.66, 0.97)	0.020	0.84 (0.74, 0.95)	0.007	0.99 (0.94, 1.03)	0.57
Factor 2 (SMs)										
Model 1	0.97 (0.86, 1.09)	0.63	1.12 (1.04, 1.21)	0.003	1.21 (1.05, 1.41)	0.010	0.98 (0.87, 1.11)	0.76	1.02 (0.99, 1.05)	0.21
Model 2	0.95 (0.84, 1.07)	0.39	1.08 (1.00, 1.16)	0.049	1.14 (0.99, 1.33)	0.078	0.98 (0.87, 1.11)	0.76	1.01 (0.99, 1.04)	0.34
Model 3	0.94 (0.83, 1.06)	0.31	1.06 (0.98, 1.15)	0.13	1.10 (0.95, 1.28)	0.20	0.96 (0.85, 1.08)	0.53	1.01 (0.98, 1.04)	0.39
Factor 3 (Ceramides and dhCers)										
Model 1	0.97 (0.87, 1.08)	0.59	1.19 (1.10, 1.29)	<0.001	1.10 (0.95, 1.27)	0.19	1.00 (0.90, 1.11)	0.98	1.01 (0.98, 1.04)	0.45
Model 2	0.97 (0.87, 1.09)	0.62	1.14 (1.05, 1.24)	0.002	1.03 (0.89, 1.20)	0.69	0.98 (0.88, 1.09)	0.66	1.02 (0.99, 1.05)	0.26
Model 3	0.97 (0.86, 1.09)	0.59	1.15 (1.06, 1.25)	0.001	1.02 (0.88, 1.19)	0.80	0.97 (0.87, 1.09)	0.61	1.01 (0.98, 1.04)	0.62
Factor 4 (GSLs)										
Model 1	1.03 (0.93, 1.14)	0.59	0.94 (0.86, 1.03)	0.17	1.01 (0.87, 1.19)	0.87	1.00 (0.90, 1.11)	0.99	0.97 (0.94, 1.00)	0.06
Model 2	1.06 (0.95, 1.18)	0.27	1.01 (0.92, 1.09)	0.91	1.03 (0.89, 1.21)	0.67	0.99 (0.89, 1.10)	0.85	0.98 (0.94, 1.01)	0.12
Model 3	1.07 (0.96, 1.20)	0.20	1.02 (0.93, 1.11)	0.69	1.04 (0.89, 1.22)	0.61	1.01 (0.90, 1.12)	0.93	0.98 (0.95, 1.01)	0.15
Factor 5 (Very-long-chain SMs and hydroxysphingomyelins)										
Model 1	0.97 (0.87, 1.08)	0.53	1.03 (0.95, 1.12)	0.43	0.84 (0.73, 0.98)	0.022	1.00 (0.89, 1.13)	0.95	1.00 (0.96, 1.03)	0.76
Model 2	1.00 (0.89, 1.12)	0.98	1.00 (0.92, 1.08)	0.90	0.86 (0.74, 0.99)	0.041	1.01 (0.89, 1.14)	0.89	1.01 (0.97, 1.04)	0.64
Model 3	0.99 (0.87, 1.12)	0.83	1.01 (0.93, 1.10)	0.82	0.87 (0.75, 1.01)	0.071	1.01 (0.90, 1.15)	0.83	1.01 (0.98, 1.04)	0.60
Factor 6 (Very-long-chain hydroxysphingomyelins)										
Model 1	1.02 (0.92, 1.12)	0.73	0.99 (0.91, 1.08)	0.80	1.02 (0.88, 1.19)	0.77	1.11 (1.00, 1.23)	0.043	1.02 (0.99, 1.06)	0.16
Model 2	1.01 (0.91, 1.12)	0.92	1.00 (0.93, 1.08)	0.97	0.98 (0.84, 1.14)	0.76	1.12 (1.01, 1.24)	0.031	1.02 (0.98, 1.05)	0.30
Model 3	0.98 (0.88, 1.09)	0.75	1.01 (0.93, 1.09)	0.86	0.97 (0.83, 1.13)	0.68	1.12 (1.01, 1.25)	0.037	1.01 (0.98, 1.05)	0.42
Factor 7 (Very-long-chain ceramides and dhCers)										
Model 1	1.07 (0.96, 1.19)	0.24	1.20 (1.11, 1.30)	<0.001	1.34 (1.16, 1.54)	<0.001	1.12 (1.01, 1.24)	0.040	1.01 (0.98, 1.04)	0.70
Model 2	1.05 (0.93, 1.18)	0.46	1.09 (1.01, 1.18)	0.028	1.22 (1.06, 1.42)	0.007	1.12 (1.00, 1.24)	0.051	1.00 (0.97, 1.03)	0.89
Model 3	1.04 (0.91, 1.18)	0.61	1.09 (1.01, 1.19)	0.032	1.23 (1.06, 1.43)	0.007	1.12 (1.00, 1.26)	0.044	1.00 (0.97, 1.03)	0.94

Data are RR (95% CI) from multivariable-adjusted log-Poisson models. dhCer, dihydroceramide; GSL, glycosphingolipid; PCA, principal component analysis; RR, relative risk; SM, sphingomyelin.
Model 1, adjusted for age, sex, region (Beijing or Shanghai), residence (urban or rural), educational attainment (0–6 years, 7–9 years, or ≥10 years), current smoking (yes or no), current alcohol drinking (yes or no), physical activity (low, moderate, or high), family history of chronic diseases (yes or no), use of lipid-lowering medication (yes or no), and BMI.
Model 2, further adjusted for triglycerides, LDL-cholesterol, and HDL-cholesterol at baseline.
Model 3, further adjusted for HOMA-IR, inflammatory markers (C-reactive protein and interleukin-6), and adipokines (adiponectin and retinol-binding protein 4) at baseline.

Table S5. Association between baseline sphingolipid scores and incident metabolic syndrome, excluding subjects with use of lipid-lowering medication (n = 1213).

	Quartiles of sphingolipid scores				p_{trend}	Per SD increment	p
	Q1	Q2	Q3	Q4			
Ceramide score; n of molecules = 10							
Model 1	1	1.14 (0.91, 1.43)	1.30 (1.04, 1.62)	1.26 (1.01, 1.57)	0.026	1.10 (1.02, 1.18)	0.018
Model 2	1	1.14 (0.91, 1.43)	1.33 (1.06, 1.67)	1.31 (1.03, 1.66)	0.017	1.10 (1.01, 1.20)	0.022
Model 3	1	1.11 (0.89, 1.40)	1.30 (1.03, 1.64)	1.30 (1.02, 1.66)	0.020	1.11 (1.02, 1.21)	0.021
dhCer score; n of molecules = 8							
Model 1	1	1.14 (0.90, 1.43)	1.09 (0.86, 1.38)	1.14 (0.91, 1.44)	0.34	1.04 (0.96, 1.13)	0.30
Model 2	1	1.17 (0.94, 1.47)	1.15 (0.91, 1.45)	1.11 (0.87, 1.42)	0.50	1.04 (0.96, 1.13)	0.35
Model 3	1	1.15 (0.92, 1.44)	1.14 (0.90, 1.44)	1.11 (0.87, 1.43)	0.47	1.05 (0.96, 1.15)	0.27
SM score; n of molecules = 6							
Model 1	1	1.01 (0.82, 1.26)	0.81 (0.64, 1.02)	0.77 (0.60, 0.99)	0.016	0.90 (0.83, 0.99)	0.027
Model 2	1	1.07 (0.87, 1.32)	0.89 (0.70, 1.13)	0.84 (0.64, 1.10)	0.14	0.93 (0.84, 1.03)	0.17
Model 3	1	1.03 (0.84, 1.27)	0.86 (0.67, 1.09)	0.82 (0.62, 1.07)	0.10	0.93 (0.84, 1.03)	0.16
SM (OH) score; n of molecules = 11							
Model 1	1	0.82 (0.67, 1.02)	0.62 (0.48, 0.80)	0.60 (0.45, 0.80)	<0.001	0.81 (0.74, 0.89)	<0.001
Model 2	1	0.93 (0.75, 1.16)	0.76 (0.58, 1.00)	0.72 (0.52, 1.01)	0.033	0.86 (0.77, 0.96)	0.008
Model 3	1	0.90 (0.72, 1.12)	0.77 (0.58, 1.01)	0.77 (0.55, 1.07)	0.084	0.87 (0.78, 0.98)	0.019
GSL score; n of molecules = 5							
Model 1	1	0.97 (0.79, 1.17)	0.71 (0.56, 0.89)	0.81 (0.63, 1.05)	0.046	0.92 (0.84, 1.01)	0.09
Model 2	1	1.04 (0.85, 1.27)	0.80 (0.63, 1.00)	0.93 (0.71, 1.21)	0.35	0.97 (0.88, 1.07)	0.58
Model 3	1	1.06 (0.87, 1.29)	0.76 (0.61, 0.97)	0.96 (0.73, 1.25)	0.41	0.99 (0.89, 1.09)	0.77

Data are RR (95% CI) from multivariable-adjusted log-Poisson models. dhCer, dihydroceramide; GSL, glycosphingolipid; RR, relative risk; SM, sphingomyelin; SM (OH), sphingomyelin with one additional hydroxyl.
Model 1, adjusted for age, sex, region (Beijing or Shanghai), residence (urban or rural), educational attainment (0–6 years, 7–9 years, or ≥10 years), current smoking (yes or no), current alcohol drinking (yes or no), physical activity (low, moderate, or high), family history of chronic diseases (yes or no), use of lipid-lowering medication (yes or no), and BMI.
Model 2, further adjusted for triglycerides, LDL-cholesterol, and HDL-cholesterol at baseline.
Model 3, further adjusted for HOMA-IR, inflammatory markers (C-reactive protein and interleukin-6), and adipokines (adiponectin and retinol-binding protein 4) at baseline.

Table S6. Association between baseline sphingolipid scores and incident metabolic syndrome components.

	Central obesity (n = 1156)		Hypertriglyceridemia (n = 1713)		Low HDL-cholesterol (n = 1312)		Elevated blood pressure (n = 702)		Hyperglycemia (n = 1345)	
	**RR (95% CI)**	***p***	**RR (95% CI)**	***p***	**RR (95% CI)**	***p***	**RR (95% CI)**	***p***	**RR (95% CI)**	***p***
Ceramide score; *n* of molecules = 10										
Model 1	0.97 (0.87, 1.07)	0.52	1.19 (1.10, 1.29)	<0.001	1.14 (0.99, 1.32)	0.076	0.99 (0.89, 1.11)	0.89	1.00 (0.97, 1.03)	0.86
Model 2	0.98 (0.87, 1.10)	0.73	1.15 (1.06, 1.25)	0.001	1.06 (0.90, 1.25)	0.51	0.96 (0.85, 1.08)	0.48	1.01 (0.98, 1.04)	0.60
Model 3	0.99 (0.87, 1.12)	0.84	1.16 (1.06, 1.26)	0.001	1.04 (0.88, 1.22)	0.66	0.95 (0.85, 1.07)	0.43	1.00 (0.97, 1.04)	0.94
dhCer score; *n* of molecules = 8										
Model 1	0.92 (0.82, 1.03)	0.13	1.15 (1.05, 1.25)	0.002	1.13 (0.96, 1.32)	0.13	1.01 (0.90, 1.13)	0.89	1.00 (0.97, 1.03)	0.85
Model 2	0.91 (0.81, 1.04)	0.17	1.14 (1.04, 1.25)	0.005	1.05 (0.89, 1.24)	0.55	0.98 (0.87, 1.10)	0.73	1.01 (0.97, 1.04)	0.71
Model 3	0.91 (0.80, 1.05)	0.19	1.14 (1.04, 1.26)	0.005	1.05 (0.88, 1.25)	0.59	0.97 (0.86, 1.10)	0.64	1.00 (0.97, 1.04)	0.85
SM score; *n* of molecules = 6										
Model 1	0.87 (0.77, 0.99)	0.033	1.04 (0.95, 1.14)	0.35	0.99 (0.83, 1.18)	0.89	0.92 (0.82, 1.04)	0.17	0.97 (0.94, 1.01)	0.14
Model 2	0.90 (0.78, 1.03)	0.11	1.08 (0.98, 1.19)	0.11	0.95 (0.79, 1.15)	0.60	0.88 (0.78, 1.00)	0.046	0.99 (0.95, 1.03)	0.67
Model 3	0.91 (0.80, 1.04)	0.16	1.07 (0.97, 1.18)	0.16	0.93 (0.77, 1.12)	0.42	0.86 (0.77, 0.97)	0.017	0.99 (0.95, 1.04)	0.68
SM (OH) score; *n* of molecules = 11										
Model 1	0.87 (0.76, 0.99)	0.041	0.93 (0.84, 1.03)	0.15	0.81 (0.68, 0.96)	0.014	0.91 (0.80, 1.03)	0.15	0.96 (0.92, 1.00)	0.07
Model 2	0.91 (0.78, 1.05)	0.20	0.97 (0.87, 1.08)	0.60	0.81 (0.67, 0.97)	0.019	0.87 (0.77, 0.99)	0.041	0.98 (0.93, 1.03)	0.42
Model 3	0.92 (0.79, 1.06)	0.25	0.98 (0.88, 1.09)	0.71	0.81 (0.67, 0.97)	0.022	0.86 (0.75, 0.98)	0.023	0.99 (0.94, 1.04)	0.59
GSL score; *n* of molecules = 5										
Model 1	0.95 (0.85, 1.07)	0.44	1.02 (0.93, 1.12)	0.62	1.05 (0.88, 1.25)	0.59	1.01 (0.90, 1.13)	0.91	0.96 (0.93, 1.00)	0.039
Model 2	0.98 (0.87, 1.11)	0.80	1.04 (0.95, 1.14)	0.40	1.02 (0.85, 1.21)	0.87	0.98 (0.87, 1.11)	0.78	0.97 (0.93, 1.01)	0.14
Model 3	0.99 (0.88, 1.13)	0.92	1.05 (0.95, 1.15)	0.34	1.00 (0.84, 1.19)	0.99	0.98 (0.87, 1.10)	0.68	0.97 (0.93, 1.01)	0.17

Data are RR (95% CI) from multivariable-adjusted log-Poisson models. dhCer, dihydroceramide; GSL, glycosphingolipid; RR, relative risk; SM, sphingomyelin; SM (OH), sphingomyelin with one additional hydroxyl.

Model 1, adjusted for age, sex, region (Beijing or Shanghai), residence (urban or rural), educational attainment (0–6 years, 7–9 years, or ≥10 years), current smoking (yes or no), current alcohol drinking (yes or no), physical activity (low, moderate, or high), family history of chronic diseases (yes or no), use of lipid-lowering medication (yes or no), and BMI.

Model 2, further adjusted for triglycerides, LDL-cholesterol, and HDL-cholesterol at baseline.

Model 3, further adjusted for HOMA-IR, inflammatory markers (C-reactive protein and interleukin-6), and adipokines (adiponectin and retinol-binding protein 4) at baseline.

Table S7. Adjusted RRs for MetS according to joint classification of inflammatory markers and adipokines.

	Quartile of ceramide score					p_{trend}	$p_{interaction}$
	n	Q1	Q2	Q3	Q4		
CRP							0.007
≤ median	621	1	1.21 (0.84, 1.75)	1.11 (0.77, 1.61)	0.80 (0.52, 1.23)	0.249	
> median	621	1	1.22 (0.93, 1.59)	1.52 (1.17, 1.98)	1.49 (1.12, 1.97)	0.002	
IL-6							0.039
≤ median	607	1	1.22 (0.88, 1.69)	1.40 (1.02, 1.91)	1.31 (0.91, 1.89)	0.121	
> median	602	1	1.15 (0.84, 1.58)	1.25 (0.91, 1.72)	1.45 (1.05, 2.00)	0.020	
Adiponectin							0.038
≤ median	604	1	1.10 (0.84, 1.42)	1.08 (0.83, 1.41)	1.04 (0.77, 1.39)	0.85	
> median	605	1	1.08 (0.72, 1.62)	1.68 (1.15, 2.46)	1.73 (1.15, 2.58)	0.001	
RBP4							0.616
≤ median	621	1	1.15 (0.81, 1.63)	1.26 (0.89, 1.79)	1.20 (0.83, 1.73)	0.335	
> median	621	1	1.23 (0.94, 1.61)	1.24 (0.93, 1.65)	1.30 (0.95, 1.76)	0.115	
	**Quartile of SM (OH) score**					p_{trend}	$p_{interaction}$
	n	Q1	Q2	Q3	Q4		
CRP							0.513
≤ median	621	1	0.83 (0.58, 1.20)	0.62 (0.40, 0.96)	0.47 (0.27, 0.81)	0.004	
> median	621	1	0.98 (0.75, 1.28)	0.91 (0.66, 1.25)	0.95 (0.65, 1.38)	0.723	
IL-6							0.344
≤ median	607	1	0.71 (0.53, 0.96)	0.55 (0.37, 0.81)	0.55 (0.35, 0.86)	0.006	
> median	602	1	0.99 (0.73, 1.33)	0.94 (0.65, 1.34)	0.95 (0.61, 1.48)	0.779	
Adiponectin							0.282
≤ median	604	1	0.91 (0.69, 1.18)	0.74 (0.53, 1.03)	0.77 (0.53, 1.12)	0.122	
> median	605	1	0.74 (0.52, 1.06)	0.79 (0.52, 1.21)	0.78 (0.46, 1.34)	0.367	
RBP4							0.493
≤ median	621	1	0.72 (0.51, 1.02)	0.64 (0.42, 0.96)	0.70 (0.41, 1.17)	0.118	
> median	621	1	0.82 (0.62, 1.08)	0.84 (0.61, 1.15)	0.75 (0.51, 1.10)	0.160	

Model was adjusted for age, sex, region (Beijing or Shanghai), residence (urban or rural), educational attainment (0–6 years, 7–9 years, or ≥10 years), current smoking (yes or no), current alcohol drinking (yes or no), physical activity (low, moderate, or high), family history of chronic diseases (yes or no), use of lipid-lowering medication (yes or no), BMI, triglycerides, LDL-cholesterol, and HDL-cholesterol at baseline. CRP, C-reactive protein; IL-6, interleukin-6; MetS, metabolic syndrome; RBP4, retinol-binding protein 4; RR, relative risk; SM (OH), sphingomyelin with one additional hydroxyl.

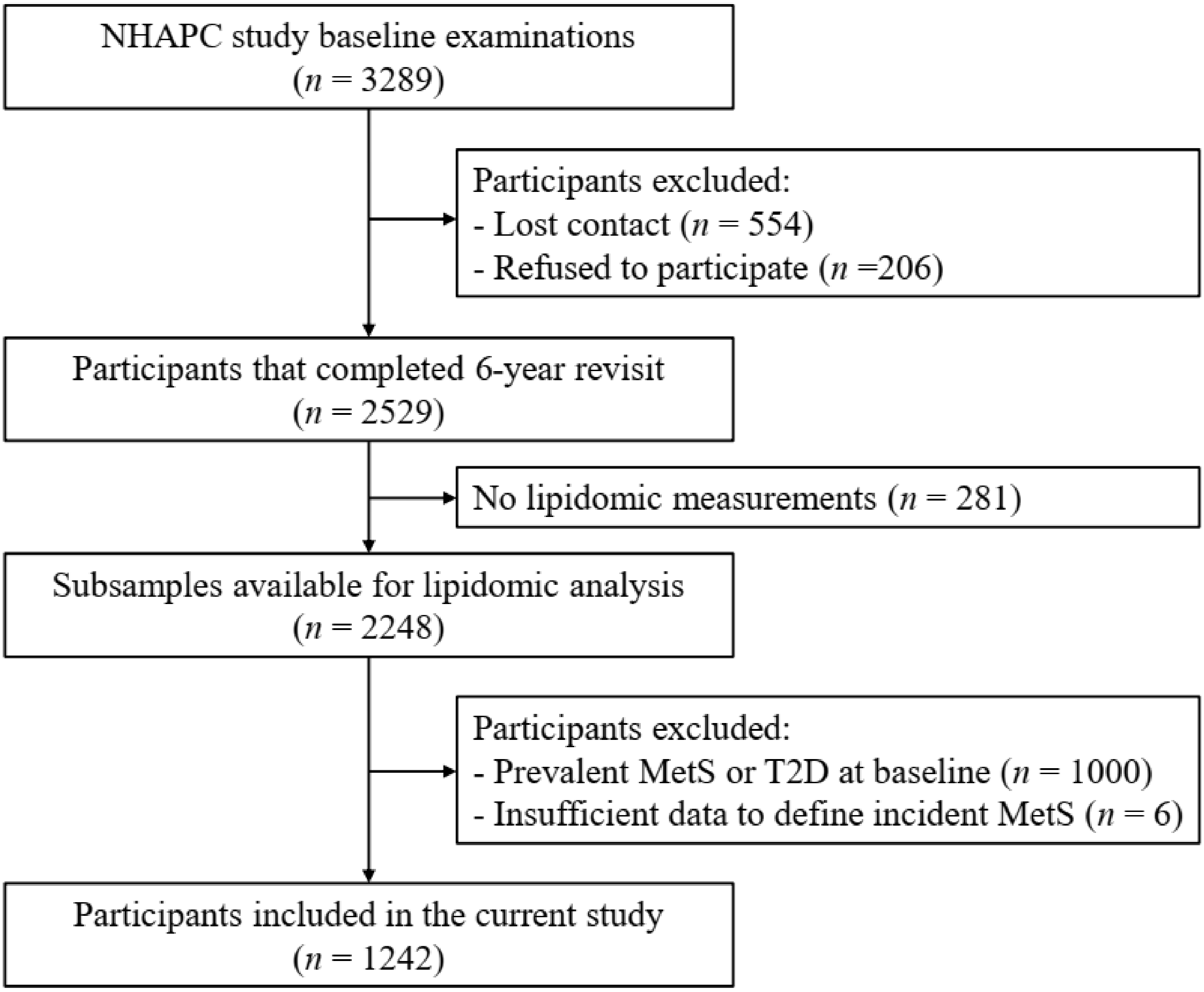

Figure S1. Flow chart of participant selection. MetS, metabolic syndrome; NHPAC, Nutrition and Health of Aging Population in China; T2D, type 2 diabetes.

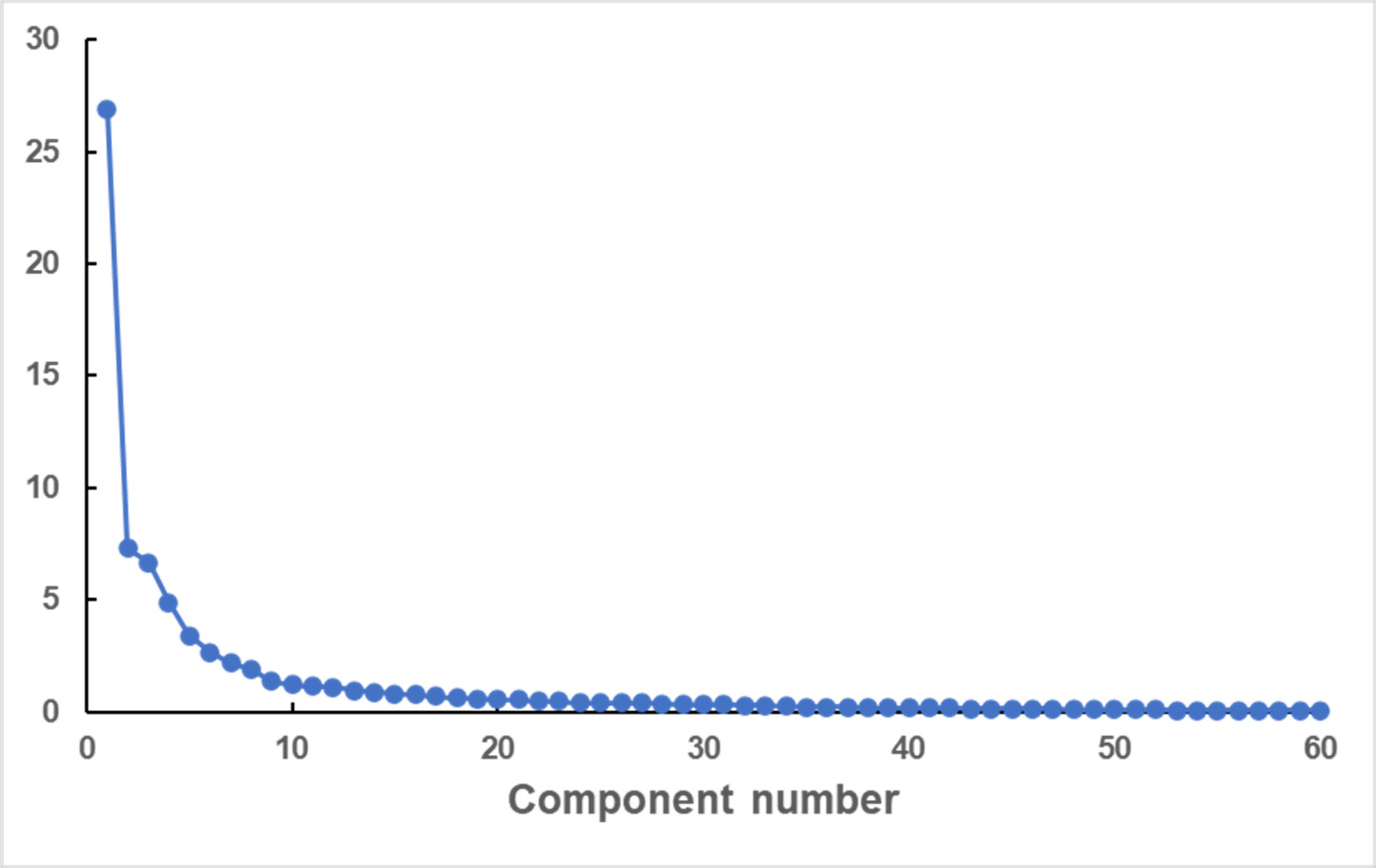

Figure S2. Scree plot of principal component analysis.

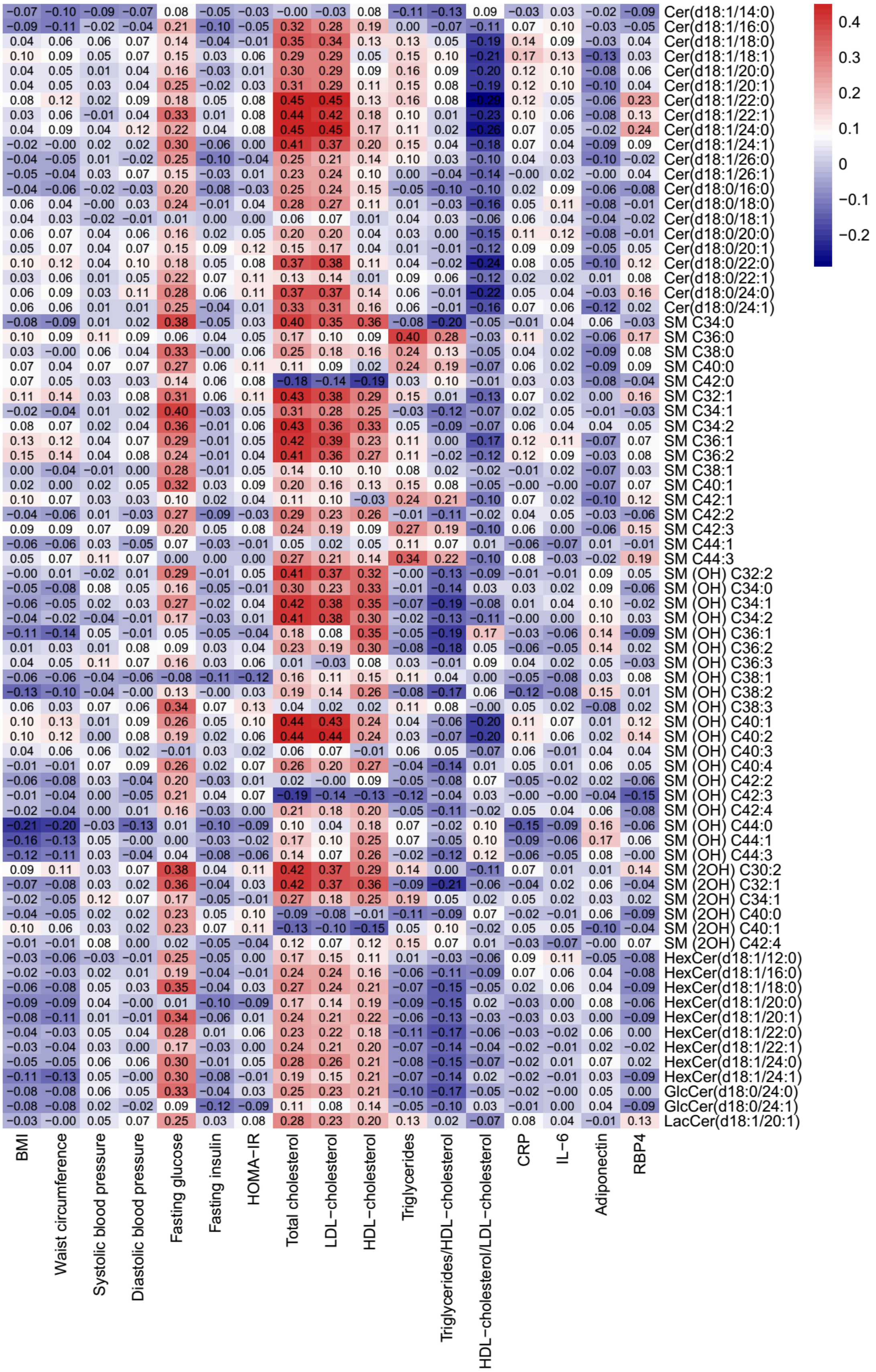

Figure S3. Spearman correlation coefficients between sphingolipids and metabolic risk factors at baseline. Values were adjusted for age, sex, region (Beijing or Shanghai), and residence (urban or rural). Cer, ceramide; CRP, C-reactive protein; dhCer, dihydroceramide; GlcCer, glucosylceramide; GSL, glycosphingolipid; HexCer, hexosylceramide; HOMA-IR, homeostatic model assessment of insulin resistance; IL-6, interleukin-6; LacCer, lactosylceramide; RBP4, retinol-binding protein 4; SM, sphingomyelin; SM (OH), sphingomyelin with one additional hydroxyl; SM (2OH), sphingomyelin with two additional hydroxyls.

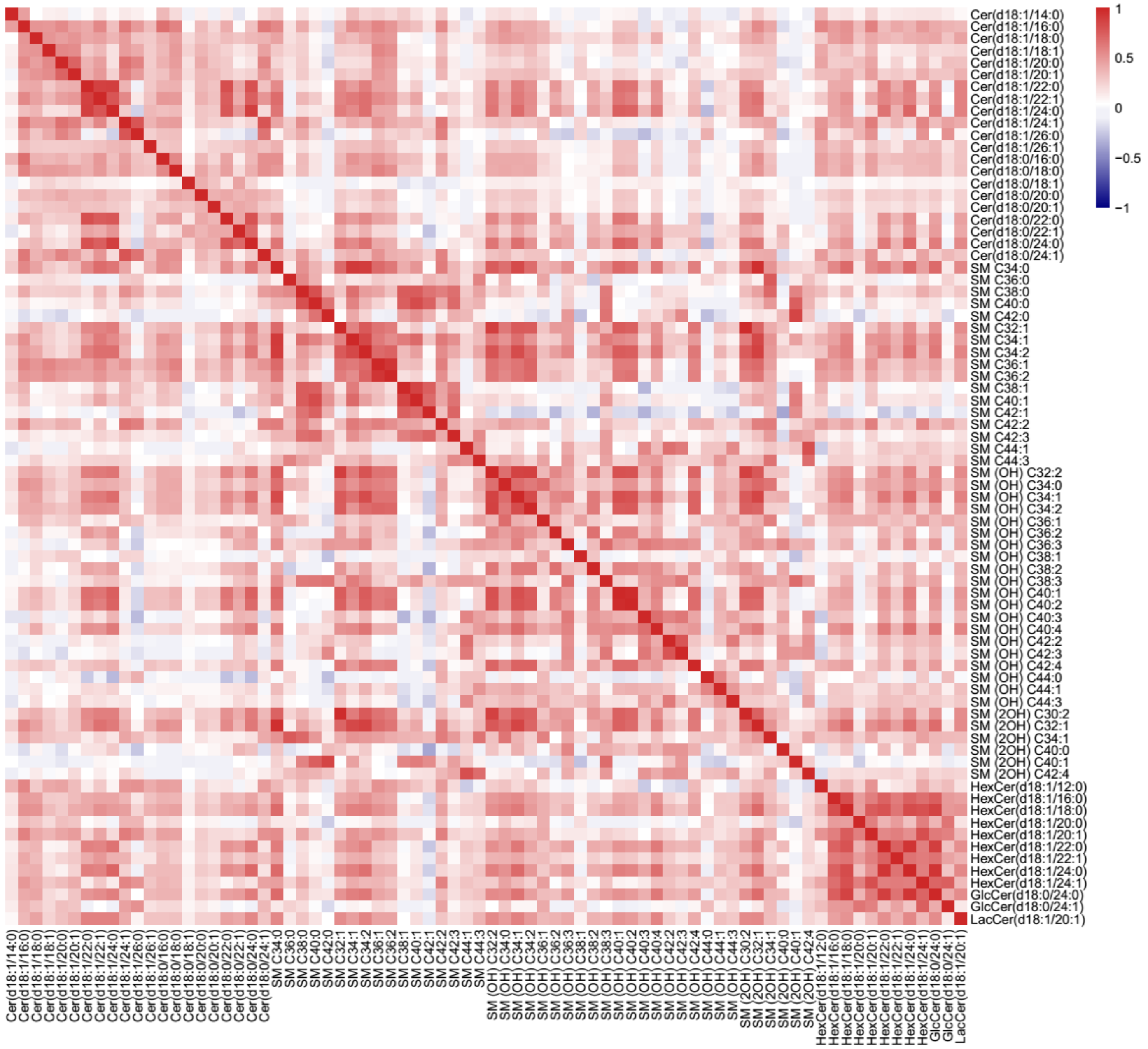

Figure S4. The correlations among baseline concentrations of sphingolipids. Cer, ceramide; dhCer, dihydroceramide; GlcCer, glucosylceramide; GSL, glycosphingolipid; HexCer, hexosylceramide; LacCer, lactosylceramide; SM, sphingomyelin; SM (OH), sphingomyelin with one additional hydroxyl; SM (2OH), sphingomyelin with two additional hydroxyls.
